# Increased gastrin-releasing peptide (GRP) receptor expression in tumour cells confers sensitivity to [Arg^6^,D-Trp^7,9^,*N*^me^Phe^8^]-substance P (6–11)-induced growth inhibition

**DOI:** 10.1038/sj.bjc.6600957

**Published:** 2003-05-27

**Authors:** C M Waters, A C MacKinnon, J Cummings, U Tufail-Hanif, D Jodrell, C Haslett, T Sethi

**Affiliations:** 1 Lung Inflammatory Group, Centre for Inflammation Research, University of Edinburgh Medical School, Hugh Robson Building, George Square, Edinburgh EH8 9XD, UK; 2 Cancer Research UK, Medical Oncology Unit, Western General Hospital, Crewe Road South, Edinburgh EH4 2XU, UK

**Keywords:** gastrin-releasing peptide, SP-G, substance P analogues, tumour growth

## Abstract

[Arg^6^,D-Trp^7,9^,*N*^me^Phe^8^]-substance P (6–11) (SP-G) is a novel anticancer agent that has recently completed phase I clinical trials. SP-G inhibits mitogenic neuropeptide signal transduction and small cell lung cancer (SCLC) cell growth *in vitro* and *in vivo*. Using the SCLC cell line series GLC14, 16 and 19, derived from a single patient during the clinical course of their disease and the development of chemoresistance, it is shown that there was an increase in responsiveness to neuropeptides. This was paralleled by an increased sensitivity to SP-G. In a selected panel of tumour cell lines (SCLC, non-SCLC, ovarian, colorectal and pancreatic), the expression of the mitogenic neuropeptide receptors for vasopressin, gastrin-releasing peptide (GRP), bradykinin and gastrin was examined, and their sensitivity to SP-G tested *in vitro* and *in vivo*. The tumour cell lines displayed a range of sensitivity to SP-G (IC_50_ values from 10.5 to 119 *μ*M). The expression of the GRP receptor measured by reverse transcriptase–polymerase chain reaction, correlated significantly with growth inhibition by SP-G. Moreover, introduction of the GRP receptor into rat-1A fibroblasts markedly increased their sensitivity to SP-G. The measurement of receptor expression from biopsy samples by polymerase chain reaction could provide a suitable diagnostic test to predict efficacy to SP-G clinically. This strategy would be of potential benefit in neuropeptide receptor-expressing tumours in addition to SCLC, and in tumours that are relatively resistant to conventional chemotherapy.

Despite recent advances in cytotoxic drug development, it is a universal goal to develop novel cancer therapies that are more specific to cancer cells and produce minimal damage to nonmalignant cells. One approach to realising this goal is the development of drugs that are able to block the growth-promoting effects of cancer cell mitogens.

Neuropeptides are important growth factors in a number of cancers including breast, colon, pancreatic, prostate, renal, gastric carcinoma and small-cell lung cancer (SCLC). SCLC cells proliferate in response to a range of neuropeptide growth factors, and in many cases these neuropeptides are involved in autocrine and paracrine growth loops that fuel unrestrained proliferation (Moody *et al*, 1985; Sethi and Rozengurt, 1991; Sethi *et al*, 1992,^1993^; North *et al*, 1997). Drugs that target specific mitogenic neuropeptides have shown little promise. Monoclonal antibodies have been developed against circulating bombesin and one such antibody, 2A11, has been shown to inhibit the growth of SCLC *in vitro* and also as xenografts in nude mice (Chaudry *et al*, 1999); however, it has limited efficacy in human trials. Thus, ‘broad-spectrum’ neuropeptide receptor antagonists have been the main focus of research in this drug development strategy. Synthetic analogues of substance P, for example, [Arg^6^,D-Trp^7,9^,*N*^me^Phe^8^]-substance P (6–11) (SP-G), were initially identified as antagonists of substance P-mediated cellular effects and were subsequently found to also antagonise the cellular effects of bombesin (Jensen *et al*, 1984). When tested in SCLC cell lines, it was found that several substance P analogues inhibited calcium mobilisation stimulated by the neuropeptides: bombesin, bradykinin, gastrin, galanin, vasopressin, cholecystokinin and neurotensin (Woll and Rozengurt, 1988; Langdon *et al*, 1992; Sethi *et al*, 1992). They were also found to inhibit mitogenesis by the same range of neuropeptides in both Swiss 3T3 cells and SCLC cells (Woll and Rozengurt, 1988; Bepler *et al*, 1989; Sethi *et al*, 1992).

In addition to the *in vitro* growth-inhibitory effects of substance P analogues, these compounds inhibit the growth of tumours in xenograft models in nude mice (Langdon *et al*, 1992; Jones *et al*, 1997). SP-G has recently completed a phase I clinical trial and will be entering a phase II clinical trial in SCLC in the near future (Clive *et al*, 2001). The exact mechanism by which these compounds exert their antitumour effects are unknown, but we have previously shown that they act as ‘biased’ agonists inhibiting neuropeptide-stimulated growth while directly stimulating apoptosis (Tallett *et al*, 1996; MacKinnon *et al*, 1999,^2001^). Understanding the exact manner in which substance P analogues modulate neuropeptide receptor signalling will allow for the rational design of more potent analogues. Identification of biological features in tumours that confer sensitivity to SP-G will elucidate the most effective use of this group of compounds in clinical practice.

In this study, we measured the expression of the mitogenic neuropeptide receptors for vasopressin (V_1A_), gastrin-releasing peptide (GRP), bradykinin (BK_2_) and gastrin by reverse transcriptase–polymerase chain reaction (RT–PCR), and show that GRP-receptor expression predicts sensitivity to SP-G in a variety of tumours (SCLC, non-small-cell lung cancer (NSCLC), colorectal, ovarian and pancreatic) *in vitro* and *in vivo*. Our results suggest that an antineuropeptide growth factor strategy may be effective in a wider range of tumours and may also be of benefit in these neuropeptide-expressing tumours, which have acquired relative resistance to conventional chemotherapeutic agents.

## MATERIALS AND METHODS

### Materials

Rat-1a cells and rat-1a cells stably expressing the mouse bombesin/GRP receptor (GRPR) (BOR-15) were established by the Imperial Cancer Research Fund (ICRF, London, UK). RPMI-1640, Dulbecco's essential Eagle's medium (DMEM), bombesin and vasopressin were from Sigma (Poole, UK). SP-G was a kind gift from Peptec (Copenhagen, Denmark). All other reagents were of the purest grade available.

### Cell culture

Stocks were maintained in RPMI-1640 (tumour cell lines) or DMEM (nontumour cell lines) supplemented with 10% (v v^−1^) fetal bovine serum (heat-inactivated at 57°C for 1 h) 50 U ml^−1^ penicillin, 50 *μ*g ml^−1^ streptomycin and 5 *μ*g ml^−1^ L-glutamine in a humidified atmosphere of 5% CO_2_ : 95% air at 37°C. For experimental purposes, cells were either grown in SITA medium (RPMI-1640 medium supplemented with 30 nM selenium, 5 *μ*g ml^−1^ insulin, 10 *μ*g ml^−1^ transferrin media supplement and 0.25% bovine serum albumin (tumour cell lines)) or with 0.1% (v v^−1^) fetal bovine serum in DMEM (nontumour cell lines). Rat-1A fibroblasts stably transfected with the mammalian bombesin receptor (BOR-15 cells) were cultured in the presence of 400 *μ*g ml^−1^ G418-sulphate.

### Growth assays

Liquid growth was determined in SITA medium in the presence or absence of mediators. Cell number was determined using a Coulter Counter (model Z1, Beckman Coulter, Bucks, UK). Colony growth was determined in 0.3% agarose in SITA medium for 21 days as described by Sethi and Rozengurt (1991).

### Determination of intracellular Ca^2+^concentration

Intracellular Ca^2+^concentration was determined using the fluorescent indicator Fura-2-tetraacetoxymethylester AME (FURA-2-AM 1 *μ*M) as described (Sethi *et al*, 1993) Ratiometric fluorescence was monitored in a Perkin-Elmer Fluorometric Spectrophotometer with dual excitation wavelengths of 340 and 380 nm and emission wavelength of 510 nm.

### Xenograft activity

Female *nu/nu* mice (6 weeks old) (ICRF, London, UK) were maintained in negative pressure isolators (Moredun Animal Research Unit, Edinburgh, UK). All xenografts used throughout were initially established by subcutaneous injection of 10^7^ cells from their respective cell lines and then maintained by serial passage of fragments of viable tumour as described previously (Langdon *et al*, 1992).

Mice were allocated into control and treatment groups containing six to eight mice. Treatment started when the xenografts reached a diameter of 3–10 mm with the first day of peptide administration designated as day 0. SP-G was dissolved in sterile distilled water at a concentration of 10 mg ml^−1^ and administered by i.p. injection in a volume of 0.1 ml per 20 g of body weight to yield a dose of 50 mg kg^−1^. The dose schedule employed was twice daily injections with an 8 h gap for a total of five injections. This schedule was chosen based largely on the pharmacokinetics of SP-G in *nu/nu* mice (Cummings *et al*, 1995). Controls received the same dose schedule of vehicle. Xenografts were measured either two or three times a week by means of calipers and tumour volume (*V*) was then calculated by the formula *V*=*π*/6*LW*^2^, where *L* is the longest diameter and *W* the diameter perpendicular to *L*. Results were expressed as a relative tumour volume (RTV) which is defi-ned simply as: *V* on day *x*/*V* on day 0, with RTV=1 on day 0. Xeno-graft experiments were repeated on two or three separate occasions.

### Semiquantitative RT–PCR

RNA was extracted using Tri-Reagent (Sigma, Poole, UK) from exponentially growing cells, which had been in SITA medium for 3–5 days and was subsequently treated with DNase1 to remove any DNA contamination. cDNA was produced from this RNA using a first-strand synthesis kit (Boehringer Mannheim, Roche, UK). PCR primers used were: GRPR sense 5′-ATCTTCTGTACAGTCAAGTCC-3′, antisense 5′-GCTTTCCTCATGGAAGGGATA-3′; V_1A_R sense 5′-TACCTGCTACGGCTTCATCTGC-3′, antisense 5′-ACACAGTCTTGAAGGAGATGGC-3′; BK_2_R sense 5′-CCTGAGTGTCATCACCTTCTGC-3′, antisense 5′-TTGATGACACGGCAGAGG-3′; receptor for gastrin (gastrinR) sense 5′-CCTATCTCCTTCATTCACTTGC-3′, antisense 5′-AGTGTGCTGATGGTGGTGTAGC-3′; *γ*-actin sense 5′-ATGGCATCGTCACCAACTGG-3′, antisense 5′-ATGACAATGCCAGTG-GTGGC-3′. Polymerase chain reaction products were run on a 1.5% agarose gel containing 1 *μ*g ml^−1^ ethidium bromide. Stored images of the gels were analysed by densitometry using Gel Base/Gel Blot software. *γ*-Actin levels were measured as an internal control. Each product was expressed relative to the levels of *γ*-actin for the same cDNA batch and for each PCR reaction.

## RESULTS

### Neuropeptide sensitivity in the longitudinal cell lines GLC14, 16 and 19

The GLC14, 16 and 19 cell lines are SCLC cell lines established from one patient during longitudinal follow-up (Berendsen *et al*, 1988). During this period, the tumour changed from sensitive to resistant to chemotherapy, and the *in vitro* sensitivity to chemotherapeutic agents reflected the clinical resistance to treatment (de Vries *et al*, 1989 and T.S. observations (results not shown)). Initially, neuropeptides (at a concentration of 100 nM) in each of the three cell lines were screened for their ability to increase [Ca^2+^]_i_. Table 1

**Table 1:** Neuropeptide Ca^2+^ mobilisation in the SCLC GLC longitudinal cell line series

**Ca^2+^ mobilising neuropeptides**	**GLC14**	**GLC16**	**GLC19**
Bombesin	6±3 (*n*=7)	25±5 9 (*n*=7)	100±15 (*n*=7)
Bradykinin	66±8 (*n*=8)	105±7 (*n*=8)	98±12 (*n*=10)
Carbamylcholine	83±11 (*n*=3)	104±18 (*n*=5)	113±25 (*n*=7)
Cholecystokinin	31±9 (*n*=4)	32±4 (*n*=3)	56±5 (*n*=4)
Neuromedin B	7±4 (*n*=4)	25±3 (*n*=3)	150±20 (*n*=4)
Neurotensin	— (*n*=3)	— (*n*=3)	43±12 (*n*=5)
Substance P	— (*n*=3)	39±6 (*n*=3)	72±10 (*n*=3)
Vasopressin	— (*n*=3)	28±8 (*n*=7)	42±9 (*n*=7)
Serum	105±8 (*n*=8)	109±15 (*n*=8)	119±19 (*n*=10)

The GLC19, 16 and 14 SCLC cell lines were loaded with 1 *μ*M Fura2AME washed and resuspended in 2 ml electrolyte solution and the cell suspension placed in a quartz cuvette and stirred continuously. Neuropeptides were added at 100 nM concentration and fluorescence was recorded continuously as described in Materials and Methods. Results are expressed as change in intracellular calcium (nM). The following peptides were also tested at 100 nM–1 *μ*M concentrations and no increase in [Ca^2+^]_i_ was ever observed: adrenocorticotrophin hormone, angiotensin 1, atrial natriuretic peptide, calcitonin, chorionic gonadotropin, dynorphin, *β*-endorphin, endothelin, epinephrine, galanin, growth hormone-releasing hormone, gastric-inhibitory peptide, glucagons 5-hydroxytryptamine, Leu-enkephalin, neuropeptide-Y, parathyroid hormone, substance K, thyrotrophin-releasing hormone.

and Figure 1A

**Figure 1:**
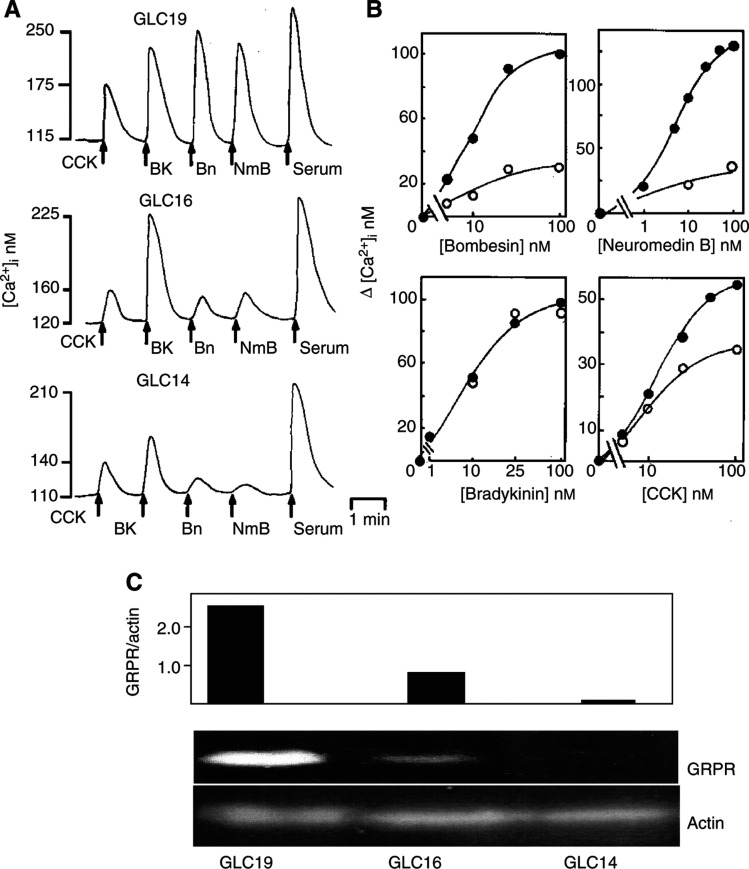
Effects of bombesin, neuromedin B, bradykinin, cholecystokinin and serum on [Ca^2+^]_i_ in SCLC cell lines GLC14, GLC16 and GLC19. Cells loaded with FURA-2-AME were resuspended in electrolyte solution and placed in a quartz cuvette. Fluorescence was monitored and basal and peak [Ca^2+^]_i_ calculated as described in Materials and Methods. Panel A: All peptides were added at a final concentration of 100 nM. Abbreviations used: BN, bombesin; NmB, neuromedin B; BK, bradykinin; CCK, cholecystokinin. Serum was added at a final concentration of 1% v v^−1^. Panel B: Dose-dependent effects of bombesin, neuromedin B, bradykinin and CCK in SCLC cell lines GLC16 and GLC19, open and closed circles, respectively. Peptides were added at the concentrations indicated. Representative concentration–response relations of three to five experiments are shown. Panel C: mRNA encoding the GRP receptor was detected by semiquantitative RT–PCR. *γ*-Actin mRNA levels were measured as an internal control for each PCR reaction. The results show a representative PCR reaction for the GLC14,16 and 19 cell lines. A bar chart showing relative receptor expression, calculated as density of PCR product/actin is shown for each cell line. The results represent the mean of two independent experiments.

show that the GLC14 cell line responded to bradykinin and serum resulting in a large increase in [Ca^2+^]_i_ and smaller responses were seen in case of neuropeptides CCK, GRP and neuromedin B. No Ca^2+^ mobilisation was observed in response to neurotensin, substance P or vasopressin. However, in the GLC19 line, addition of neuropeptides bradykinin, CCK, GRP, neuromedin B and substance P caused large increases in [Ca^2+^]_i_. Vasopressin and neurotensin caused smaller but consistent increases in [Ca^2+^]. The GLC19 was the only cell line in which neurotensin caused a measurable increase in [Ca^2+^]_i_.

One percent serum caused a rapid increase in [Ca^2+^]_i_ in all three cell lines of 100–150 nM, suggesting that the mobilisable Ca^2+^ pools were equivalent in each line; however bombesin, neuromedin B and CCK showed an increased responsiveness in the GLC19 cells. Typical concentration response curves in GLC16 and 19 cells are shown in Figure 1B. Thus, during the tumour progression from GLC14, 16 to 19 cell lines, there is an increase in the number of neuropeptides able to induce a measurable increase in [Ca^2+^]_i_ and also an increase in the potency of individual neuropeptides. The expression of the GRP receptor was examined in the GLC cell lines by RT–PCR (Figure 1C). GRP receptor expression increased by six-fold in the GLC16 compared to the GLC14 cell line and by 11-fold in the GLC19 cell line, paralleling the increased responsiveness to GRP-induced Ca^2+^ release.

**Figure 2:**
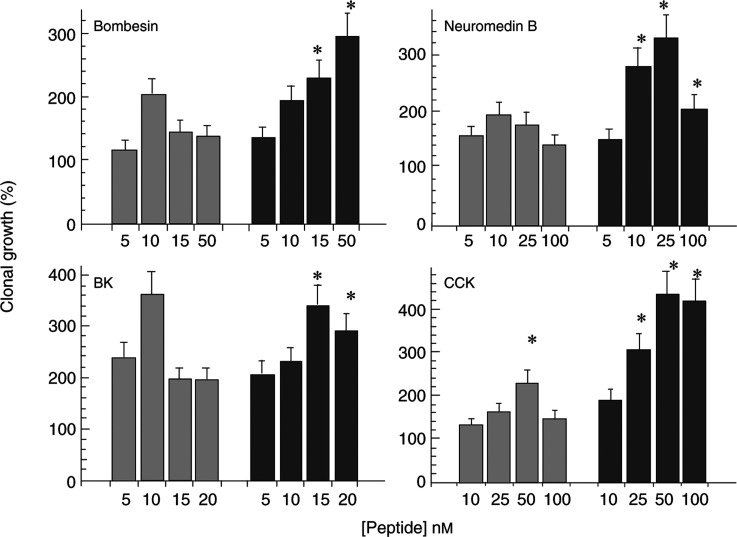
Dose-dependent effects of bombesin, BK, CCK and neuromedin B on colony formation in GLC16 and GLC19 SCLC cells. GLC16 (hatched bars) and GLC19 (filled bars) cells, 3–5 days postpassage, were washed and 10^4^ viable cells per ml were plated in SITA medium containing 0.3% agarose on top of a base of 0.5% agarose in culture medium as described in Materials and Methods. Both layers contained additions at the nanomolar concentrations indicated. Cultures were incubated at 37°C in a humidified atmosphere at 5% CO_2_ : 95% air for 21 days and then stained with nitro-tetrazolium blue. Spontaneous colony formation, that is, in the absence of any exogenously added peptide was 140±15 (*n*=75) and 160±20 (*n*=85), in the cell lines GLC16 and 19, respectively, and normalised to 100%. Each point represents the mean±s.e.m. of two to four experiments each of five replicates. ^*^Statistically different from neuropeptide concentration matched GLC16 cells (ANOVA, *P*<0.05).

CCK, bradykinin, neuromedin B and bradykinin stimulated a concentration-dependent increase in clonal growth in the GLC16 and 19 cell lines. There was a significant increase in the ability of bombesin, neuromedin B and CCK to stimulate clonal growth in the GLC19 SCLC cell line compared to the GLC16 cell line (Figure 2

). Bradykinin was equally effective in stimulating clonal growth in both GLC16 and 19 cell lines. The ability of these neuropeptides to stimulate clonal growth in the GLC14 cell line was consistently less than that seen in the GLC16 cell line. Serum stimulated a 405–370% increase in clonal growth in the GLC19, 16 and 14 cell lines, and the cloning efficiency of all three SCLC cell lines was approximately 1.5%. Hence, the GLC19 cell line develops increased Ca^2+^ responsiveness to the neuropeptides CCK, bombesin and neuromedin B, and this correlates with an increase in the ability of these neuropeptides to stimulate clonal growth.

**Figure 3:**
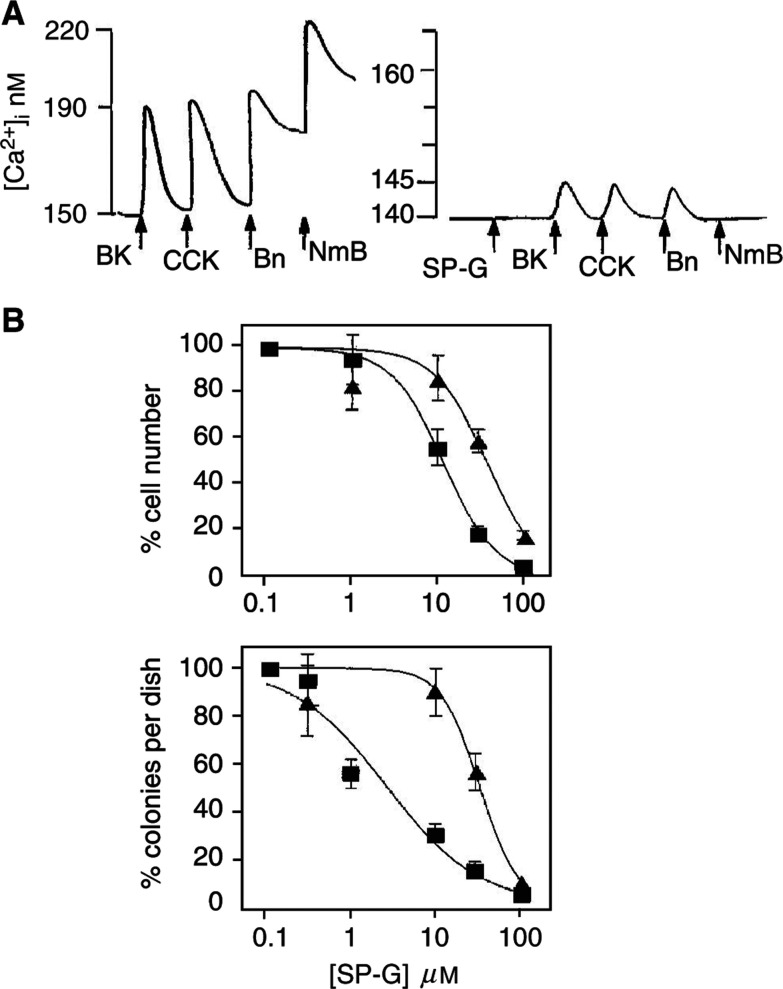
(**A**) Effect of SP-G on neuropeptide-induced Ca^2+^ mobilisation in GLC19 SCLC cells. Cells loaded with FURA-2-AME were resuspended in electrolyte solution and placed in a quartz cuvette. Fluorescence was monitored and basal and peak [Ca^2+^]_i_ calculated as described in Materials and Methods. Peptides were added at a final concentration of 30 nM and SP-G at a final concentration of 30 *μ*M. (**B**) Effect of SP-G on GLC16 and GLC19 cell growth. Liquid growth (upper panel): GLC16 (triangles) and GLC19 (squares) SCLC cells were resuspended at a density of 5 × 10^4^ cells in 1 ml SITA in the presence or absence of SP-G in triplicate. The controls (cells in absence of antagonist) after an initial lag period grew exponentially and then reached a plateau after 10 days (GLC19: 4.8±0.5 × 10^5^ (*n*=6) and GLC16: 5.7±0.5 × 10^5^ (*n*=6)). Each point represents the mean cell number after 10 days±s.e.m. of two experiments each of three replicates. Colony growth (lower panel): GLC16 (triangles) and GLC19 (squares) SCLC cultures were washed, and 10^4^ viable cells/ml were plated in SITA medium containing 0.3% agarose on top of a base of 0.5% agarose in culture medium as described in Materials and Methods. Both layers contained additions as indicated. Cultures were incubated at 37°C in a humidified atmosphere at 5% CO_2_ : 95% air for 21 days and then stained with nitro-tetrazolium blue. Each point represents the mean±s.e.m. of two experiments each of five replicates.

SP-G inhibited Ca^2+^ mobilisation induced by CCK, bombesin, neuromedin B and bradykinin in the GLC19 SCLC cell line (Figure 3A

). SP-G also inhibited the growth and cloning efficiency of the GLC14 (not shown) 16 and 19 cells, but was most potent in the GLC19 cell line (IC_50_ for the GLC14, 16 and 19 SCLC cells was 25, 25 and 15 *μ*M, respectively, for liquid culture, and 25, 25 and 5 *μ*M, respectively, for cloning efficiency, Figure 3B). Interestingly, SP-G was still effective in inhibiting the growth of the GLC19 SCLC line despite the development of resistance to conventional chemotherapeutic agents (de Vries *et al*, 1989). These findings suggest that increased neuropeptide receptor expression results in increased sensitivity to SP-G.

### Sensitivity to SP-G in a panel of tumour cell lines

A panel of tumour cell lines was selected that represents a spectrum of tumour types, NSCLC, ovarian, pancreatic and colon cancer, which express neuropeptide receptors in addition to SCLC. The characteristics of these cell lines and their sensitivity to SP-G are shown in Table 2

**Table 2:** Growth inhibition by SP-G (IC_50_) and neuropeptide receptor expression in a panel of tumour cell lines

		**Relative NP receptor expression**			
**Cell line**	**IC50 (*μ*M)**	GRPR^*^	**VA_1_R**	**BK_2_R**	**Gastrin R**
H69 (SCLC, Moody *et al*, 1985)	10.5	0.91±0.15	0.8±0.18	0.65±0.31	0.56±0.49
HT29 (colorectal, Brattain *et al*, 1981)	18	0.55±0.12	0.02±0.01	0.35±0.04	—
H510 (SCLC, Moody *et al*, 1985)	29	0.3±0.11	1.10±0.21	0.51±0.17	1.51±40.61
PEO4 (ovarian, Lieber *et al*, 1975)	31	1.31±0.25	1.10±0.02	0.24±0.10	0.2±0.05
NX002 (NSCLC, Stark *et al*, 2001)	33.5	1.01±0.21	—	0.65±0.23	0.56±0.28
HRT18 (colorectal, Fogh *et al*, 1977)	37	0.18±0.04	—	0.80±0.21	0.03±0.01
W X330 (SCLC, Hay *et al*, 1991)	42	0.09±0.01	3.71±0.91	0.22±0.12	0.27±0.18
PANC1 (pancreatic, O'Hara *et al*, 1986)	58	0.09±0.01	0.10±0.02	0.38±0.11	—
HCT116 (colorectal, Langdon *et al*, 1988)	129	—	—	0.65±0.25	—

RT–PCR products were analysed by densitometry and the relative expression level of four neuropeptide receptors are summarised. These values have been expressed as a ratio of the level of *γ*-actin expression for each cDNA batch and PCR run. Numbers represent the mean of four to six experiments. Spearman's rank correlation between IC_50_ value and receptor expression level was performed. A significant correlation between sensitivity to SP-G and GRPR expression was confirmed (Spearman's *R* value=−0.75, *P*=0.026^*^).

. Growth inhibition by SP-G was not confined to tumour type. The two most sensitive cells were the H69 SCLC cell line (IC_50_=10.5 *μ*M) and the HT29 colorectal cell line (IC_50_=18 *μ*M). Four out of five SCLC cell lines had IC_50_ values below the mean IC_50_ value of 44 *μ*M. Both the ovarian carcinoma and the non-small-cell carcinoma were sensitive to growth inhibition by SP-G, with IC_50_ values of 31 and 33.5 *μ*M, respectively. During the phase 1 clinical trial of SP-G, plasma levels of up to 40 *μ*M were achievable with no dose-limiting toxicity (Clive *et al*, 2001). A total of 72% of the cancer cell lines tested had IC_50_ values below or near this concentration. This suggests that ‘broad–spectrum’ neuropeptide antagonists such as SP-G may be effective antitumour agents in a variety of cancers other than SCLC.

### *In vivo* sensitivity to SP-G in a panel of tumour cell lines

**Figure 4:**
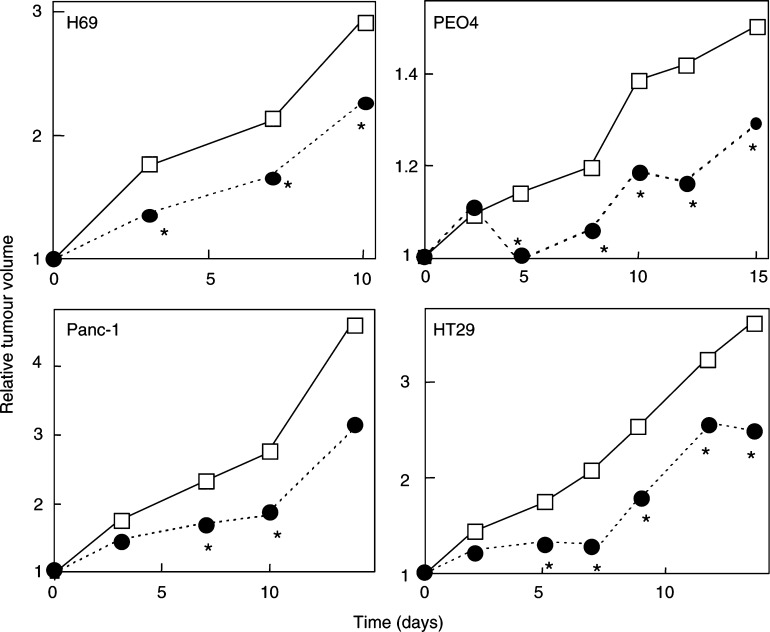
Effect of SP-G on the growth of H69, HT29, PE04 and PANC-1 xenografts. Four cell lines from the original panel were established as xenografts from their respective cell lines: H69 SCLC, HT29 colon carcinoma, PEO4 ovarian cancer and PANC-1 pancreatic cancer. Typical growth curves are shown for each xenograft after i.p. treatment with 50 mg kg^−1^ SP-G (•) or vehicle control (□) as five separate injections administered over 3 days as two injections on day 1 and 2 separated by a 6-h gap with the final injection given on day 3. The results represent the mean tumour volumes from a single study (*n*=6–8). ^*^Statistically significant from vehicle control (Students' *t*-test, *P*<0.05).

Four members from the original panel of tumour cell lines were established as xenografts from their respective cell lines: H69 SCLC, HT29 colon carcinoma, PEO4 ovarian cancer and PANC-1 pancreatic cancer. Typical growth inhibition curves (Figure 4

) are shown for each xenograft after i.p. treatment with 50 mg/kg SP-G. The HT29 xenograft showed a growth inhibition of 70.4%, 7 days after the commencement of treatment. In addition, this growth inhibition was maintained over 17 days after the final injection (Figure 4). The PEO4 ovarian xenograft showed a maximum growth inhibition of 69.3% at day 7, and in common with HT29, growth inhibition was also maintained over 17 days beyond the final injection. The H69 xenograft showed a maximum growth inhibition of 39.9% to SP-G that was sustained for 10 days after treatment. The PANC-1 pancreatic tumour cell line was one of the least-sensitive cells lines to SP-G *in vitro* and proved to be the least-sensitive xenograft, with statistically significant growth inhibition recorded at only two time points, day 7 and day 10 (Figure 4). Overall, these data report, for the first time, that SP-G has marked *in vivo* xenograft activity in a variety of tumours including lung, colon, ovarian and pancreatic cancer, and in cancers such as ovarian cancer (with *in vitro* derived chemoresistance), NSCLC and colon cancer, which are relatively insensitive to standard conventional chemotherapy.

### Correlation of neuropeptide receptor expression with sensitivity to SP-G in a panel of tumour cell lines

**Figure 5:**
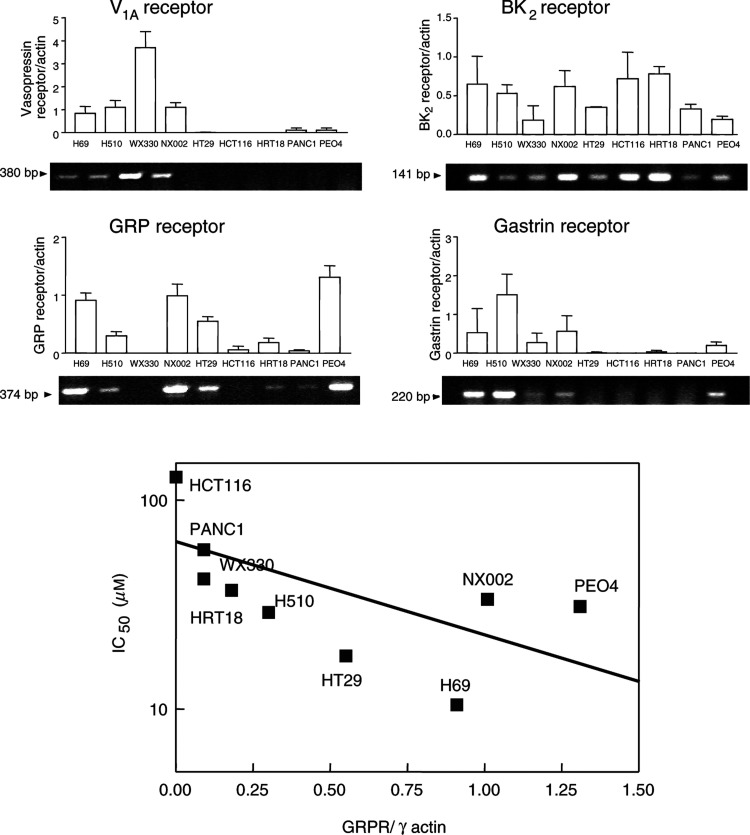
Upper panel: Correlation between neuropeptide receptor expression and sensitivity to SP-G in tumour cell lines. mRNA encoding the GRP, V_1A_, BK_2_ and gastrin receptors was detected by semiquantitative RT–PCR. *γ*-Actin mRNA levels were measured as an internal control for each PCR reaction. The results show a representative PCR reaction for each of nine tumour cell lines, which represent a spectrum of sensitivity to SP-G. A bar chart showing relative receptor expression, calculated as density of PCR product/actin is shown for each receptor. The results represent the mean±s.e.m. of four independent experiments. Lower panel: Correlation graph between GRPR expression and inhibition of cell growth (IC_50_) for each of nine tumour cell lines as indicated (Spearman's *R* value=−0.75, *P*=0.026^*^).

The expression of GRP, gastrin, BK_2_ and V_1A_ receptors were examined by RT–PCR, in nine cell lines from the original panel representing a spectrum of sensitivity to SP-G. The results are shown in Table 2 and Figure 5

(*n*=4–5 independent experiments). Receptors for vasopressin (V_1A_R) were detected in four out of four lung cancer cell lines (three SCLC and one NSCLC). The WX330 SCLC cell line was the highest expresser of V_1A_R. The pancreatic and ovarian cancer lines expressed low levels of the V_1A_R and in the colorectal cancer cell lines only HT29 cells expressed V_1A_R. GRPRs were detected in three out of four lung cancer cell lines (two SCLC and one NSCLC). In the colorectal cell lines, the HT29 cell line showed high levels of GRPR expression and HCT116 cell line showed no detectable GRPR expression with intermediate expression in the HT18 cell line. The pancreatic cell line PANC1 had very low levels of GRPR expression. The PEO4 ovarian cancer cell line and H69 SCLC cell lines showed high expression of the GRPR (Table 2). Receptors for bradykinin were detected in all cells of the cell panel. Other studies have found an almost ubiquitous expression of bradykinin receptors in human lung cancers (Bunn *et al*, 1992). Gastrin receptors were detected in three out of four lung lines (two SCLC and one NSCLC) and low expression was detected in the ovarian line PEO4. The highest expression was in the SCLC cell line H510. Interestingly, none of the colorectal cell lines expressed gastrin receptors (Table 2). This finding confirms other studies, which show that the gastrin receptor is rarely expressed in colorectal cancer cell lines (Reubi *et al*, 1997). These results show that sensitivity to SP-G in a variety of tumours appears to correlate with the level of expression of neuropeptide receptors. In particular, of all the neuropeptide receptors examined, the correlation between relative GRP receptor expression (receptor/*γ*-actin) and sensitivity to SP-G was the most apparent (Spearmans correlation *R*=−0.75; *P*=0.026, Figure 5).

### Effect of GRP receptor expression on SP-G sensitivity in fibroblasts

**Figure 6:**
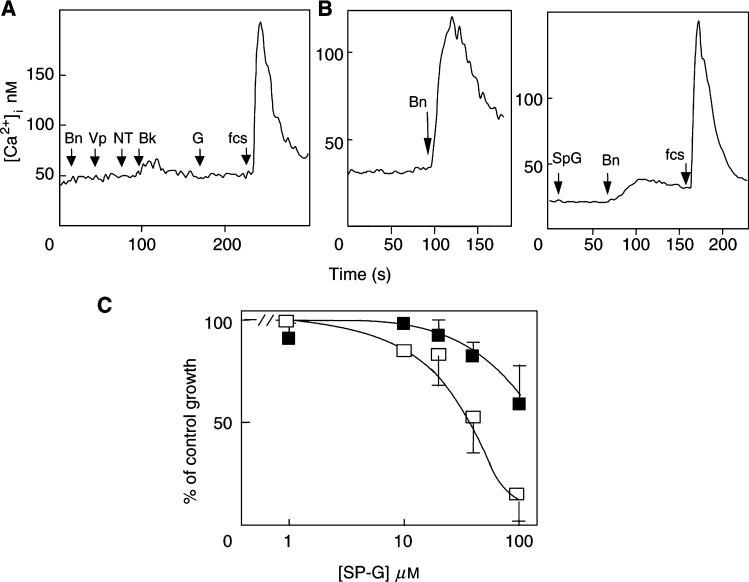
Expression of the bombesin/GRP receptor in Rat-1 fibroblasts increases sensitivity to growth inhibition by SP-G. Rat-1a fibroblasts (**A**) or rat-1a fibroblasts expressing the bombesin receptor (**B**) were loaded with FURA-2-AME and resuspended in electrolyte solution. Fluorescence was monitored and basal and peak [Ca^2+^]_i_ calculated as described in Materials and Methods. (**A**) Rat-1a cells were stimulated with 10 nM each of bombesin (Bn), vasopressin (Vp), neurotensin (NT) and bradykinin (Bk), 10 *μ*M SP-G or 1% FCS. The results are representative of three experiments. (**B**) (left) Response to 10 nM bombesin in BOR-15 cells and (right) effect of 10 *μ*M SP-G on bombesin-stimulated Ca^2+^ release in BOR-15 cells. (**C**) Rat-1 fibroblasts (closed squares) or Rat-1 fibroblasts expressing the bombesin receptor (BOR15, open squares) were grown in SITA media in the presence or absence of the indicated concentrations of SP-G. The cell number was determined and IC_50_ values calculated following 7 days of growth.

The importance of neuropeptide receptor status for cell sensitivity to SP-G was further tested in a rat-1 fibroblast system in which only one neuropeptide receptor (the mouse GRPR) was expressed at high levels (*K*_d_=1 nM, *B*_max_=10^5^ receptors per cell) (Charlesworth *et al*, 1996). In native rat-1a fibroblasts, bombesin and other Ca^2+^-mobilising neuropeptides, vasopressin, neurotensin, bradykinin and gastrin, failed to mobilise intracellular Ca^2+^, suggesting absence of receptors in the parent cell line (Figure 6A

). Rat-1a fibroblast cells stably expressing the mammalian bombesin receptor (BOR15) respond normally to bombesin stimulation in terms of signal transduction and stimulation of DNA synthesis (Charlesworth *et al*, 1996; MacKinnon *et al*, 2001). Bombesin stimulated a marked and rapid increase in [Ca^2+^]_i_ in BOR15 cells, which was blocked by Sp-G (Figure 6B). The effect of SP-G on the growth of rat-1a fibroblasts and BOR15 cells was therefore determined. The parent rat-1a cells were comparatively resistant to SP-G-mediated growth inhibition with only partial inhibition observed at the highest concentration of SP-G (41+12% inhibition at 100 *μ*M, IC_50_>100 *μ*M), whereas BOR15 cell growth was completely inhibited by 80 *μ*M SP-G (IC_50_ 44 *μ*M, Figure 6). These results show that the presence of the bombesin receptor leads to increased sensitivity to growth inhibition by SP-G in fibroblasts.

## DISCUSSION

The GLC14, 16 and 19 are classic type SCLC cell lines derived from a 55-year-old female with SCLC. The GLC14 cell line was established from a supraclavicular lymph node before treatment. After chemotherapy, the patient was in complete remission, 4 months later she relapsed. Further chemotherapy resulted in a partial response and the GLC16 line was established from a recurrence in the lung. After radiotherapy, the lung appeared tumour free; however, 3 months later tumour recurred in the lung from which the GLC19 cell line was derived. This was resistant to any further treatment and the patient died 2 months later (Berendsen *et al*, 1988; de Vries *et al*, 1989). *In vitro* sensitivity to chemotherapeutic agents reflected the clinically observed development of resistance to treatment (de Vries *et al*, 1989). We were also able to confirm these findings in the GLC cells used in this study (results not shown). One percent serum caused a rapid and equivalent increase in [Ca^2+^]_i_ in all three GLC SCLC cell lines suggesting that the mobilisable Ca^2+^ pools were equivalent in each of the three cell lines. However, we show that in the progression to chemoresistance the GLC cells are able to respond to a greater range of neuropeptides with increased potency, and are consequently more sensitive to growth inhibition by SP-G. The GLC19 cell line was more sensitive to SP-G both in liquid culture and in semisolid medium than the GLC16 and 14 cell lines. The Ca^2+^-mobilisation and the clonal growth results suggest that this cell line may have greater neuropeptide dependence and this is reflected in its greater sensitivity to neuropeptide growth factor blocking agents such as SP-G. This led to the hypothesis that other neuropeptide-expressing tumours may also be sensitive to the growth-inhibitory effect of SP-G.

It is well known that neuropeptides can stimulate the growth of many types of cancers including pancreatic, colorectal, prostate, ovarian, breast and NSCLC (Bologna *et al*, 1989; Halmos *et al*, 1995; Aprikian *et al*, 1996; Ferris *et al*, 1997). A panel of 11 tumour cell lines, which represented a spectrum of tumour types, showed that sensitivity to SP-G differed across the panel with a range of >1 log order (10 *μ*M to >100 *μ*M). The sensitive cell lines (four SCLC, two colorectal carcinoma, one NSCLC and 1 ovarian) had IC_50_ values that were in the range of physiologically obtainable plasma concentrations. Moreover, in the cell lines tested *in vitro* sensitivity correlated with sensitivity to SP-G *in vivo*. The results of this screen suggest that SP-G could be used therapeutically against several tumour types in addition to SCLC.

We have previously shown that SP-G augments etoposide-induced growth inhibition and apoptosis in SCLC cells and suggested that SP-G may be of increased benefit in patients following relapse or in conjunction with conventional chemotherapy (MacKinnon *et al*, 1999). It is of interest to note that the cell lines SCLC GLC19, ovarian cancer PEO4 and NSCLC NX022, which *in vitro* (and *in vivo*) are resistant to standard chemotherapeutic agents such as etoposide, are sensitive to the growth-inhibitory effects of SP-G. This raises the possibility that SP-G may be an effective anticancer agent in patients with neuropeptide-expressing tumours which are intrinsically resistant or have acquired resistance to conventional chemotherapy. A plausible extension of this hypothesis is that clinically aggressive drug-resistant SCLC cells that emerge after chemotherapy might have a more extensive network of neuropeptide regulation and therefore display increased sensitivity to neuropeptide antagonists. Further studies in longitudinal cell lines are required to investigate this hypothesis in further detail.

Although our data cannot indicate the expression of fully functional receptors, the RT–PCR approach was taken as it could be used as a possible diagnostic test in cancer patients to determine the potential tumour sensitivity to neuropeptide growth factor antagonist therapy. Of the four neuropeptide receptors tested, the most apparent correlation was between high expression of the GRP receptor and increased sensitivity to SP-G. This is in some ways surprising given that SP-G is more selective for the V_1A_R (Seckl *et al*, 1995) which is expressed on many lung cancer cells. However, GRP secretion and GRPR expression are the hallmarks of the neuroendocrine phenotype. The presence of the GRPR in particular may be a reflection of the general neuroendocrine phenotype of the cancer cells indicating greater neuropeptide dependence for growth. It would be interesting to look at the expression of other bombesin-like peptide receptors such as the neuromedin B receptor to assess its role in substance P-analogue-induced growth inhibition.

The GRPR has been shown to be oncogenic when transfected into the nonmalignant NCM460 colon epithelial cell line (Ferris *et al*, 1997). The increased proliferation was shown to be because of constitutive activation of the GRPR in that the receptors tonically coupled to G_q_ in the absence of ligand. This gives a potential mechanism whereby the GRPR may act as an oncogene. In addition, many tumour types such as breast cancer (Halmos *et al*, 1995) and prostate cancer (Bologna *et al*, 1989; Aprikian *et al*, 1996) have also been shown to aberrently express GRPRs. We and others have previously shown that the expression of GRPRs in fibroblasts increases the ability of SP-G and other substance P analogues to activate the extracellular-signal-regulated kinase (ERK) and c-*jun*-N-terminal kinase (JNK) pathways leading to growth arrest and apoptosis (Jarpe *et al*, 1998; MacKinnon *et al*, 2001). Together, these data demonstrate that not only can GRPR expression transform cells, but these cells then become more sensitive to substance P-analogue-induced cell death.

These findings have important implications for the design of more advanced phase human clinical trials using substance P analogues. SP-G is currently entering a phase II clinical trial where its effectiveness will be tested in SCLC patients, but ultimately, compounds of this type may also be suitable for the treatment of a wide range of other tumour types and neuropeptide-expressing tumours that have become resistant to standard conventional chemotherapeutic agents. Screening tumour biopsy samples for neuropeptide receptor expression may provide insight into the likelihood of patients responding to treatment with substance P analogues, analogous to oestrogen receptor expression conferring efficacy to tamoxifen in breast cancer. It is suggested that tumours should be biopsied to select patients for substance P-analogue trials based on the expression of the GRPR and another neuropeptide receptor. Our results suggest that these tumours should show growth inhibition regardless of intrinsic or acquired resistance to standard chemotherapeutic agents. It is therefore proposed that in the first instance, patients with tumours that express the GRPR and another neuropeptide receptor who have failed conventional treatment should be randomised into two groups – best supportive care and treatment with substance P analogues.

## References

[bib1] Aprikian AG, Han K, Chevalier S, Bazinet M, Viallet J (1996) Bombesin specifically induces intracellular calcium mobilisation via gastrin-releasing peptide receptors in human prostate cancer cells. J Mol Endocrinol 16: 297–306 8782088 10.1677/jme.0.0160297

[bib2] Bepler G, Bading H, Heimann B, Kiefer P, Havemann K, Moelling K (1989) Expression of p64c-myc and neuroendocrine properties define three subclasses of small cell lung cancer. Oncogene 4: 45–50 2536917

[bib3] Berendsen HH, de-Leiji L, de Vries EG, Mesander G, Mulder NH, De-Jong B, Postmus PE, Poppema S, Sluiter HJ, The TH (1988) Clinical characterisation of three small cell lung cancer cell lines established from one patient during longitudinal follow up. Cancer Res 48: 6891–6899 2846164

[bib4] Bologna M, Festuccia C, Muzi P, Biordi L, Ciomei M (1989) Bombesin stimulates growth of human prostatic cancer cells *in vitro*. Cancer 63: 1714–1720 2539244 10.1002/1097-0142(19900501)63:9<1714::aid-cncr2820630912>3.0.co;2-h

[bib5] Brattain MG, Fine WD, Khaled FM, Thompson J, Brattain DE (1981) Heterogeneity of malignant cells from a human colonic carcinoma. Cancer Res 41: 1751–1756 7214343

[bib6] Bunn PAJ, Chan D, Dienhart DG, Tolley R, Tagawa M, Jewett PB (1992) Neuropeptide signal transduction in lung cancer: clinical implications of bradykinin sensitivity and overall heterogeneity. Cancer Res 52: 24–31 1309227

[bib7] Charlesworth A, Broad S, Rozengurt E (1996) The bombesin/GRP receptor transfected into Rat-1 fibroblasts couples to phospholipase C activation, tyrosine phosphorylation of p125FAK and paxillin and cell proliferation. Oncogene 12: 1337–1345 8649836

[bib8] Chaudry A, Carrasquillo JA, Avis IL, Shuke N, Reynolds JC, Bartholomew R, Larson SM, Cuttitta F, Johnson BE, Mulshine JL (1999) Phase I and imaging trial of a monoclonal antibody directed against gastrin-releasing peptide in patients with lung cancer. Clin Cancer Res 5: 3385–3393 10589749

[bib9] Clive S, Webb DJ, MacLellan A, Young A, Byrne B, Robson L, Smyth JF, Jodrell DI (2001) Forearm blood flow and local responses to peptide vasodilators: a novel pharmacodynamic measure in the phase I trial of antagonist G, a neuropeptide growth factor antagonist. Clin Cancer Res 10: 3071–3078 11595697

[bib10] Cummings J, MacLellan AJ, Jones DA, Langdon SP, Rozengurt E, Ritchie AA Smyth JF (1995) Pharmacokinetics, metabolism, tissue and tumour distribution of the neuropeptide growth factor antagonist [Arg6, D-Trp7,9,*N*^me^Phe8]-substance P(6–11) in nude mice bearing the H69 small-cell lung cancer xenograft. Ann Oncol 6: 595–602 8573540 10.1093/oxfordjournals.annonc.a059250

[bib11] de Vries EG, Meijer CJ, Timmer BH, Berendsen HH, Mulder NH (1989) Resistance mechanisms in three SCLC cell lines established from one patient during clinical follow up. Cancer Res 49: 4175–7178 2545337

[bib12] Ferris HA, Carroll RE, Rasenick MM, Benya RV (1997) Constitutive activation of the gastrin-releasing peptide receptor expressed by the non-malignant human colon epithelial cell line NCM460. J Clin Invest 100: 2530–2537 9366567 10.1172/JCI119795 PMC508453

[bib13] Fogh J, Fogh JM, Orfeo T (1977) One hundred and twenty-seven cultured human tumour cell lines producing tumours in nude mice. J Natl Cancer Inst 59: 221–226 327080 10.1093/jnci/59.1.221

[bib14] Halmos G, Wittliff JL, Schally AV (1995) Characterization of bombesin/gastrin-releasing peptide receptors in human breast cancer and their relationship to steroid receptor expression. Cancer Res 55: 280–287 7812958

[bib15] Hay FG, Duncan LW, Leonard RC (1991) Establishment and characterisation of two new small cell lung cancer cell lines-one from a patient with previous familial retinoblastoma. Br J Cancer 14 (Suppl): 43–45 PMC2204113 1645570

[bib16] Jarpe MB, Knall C, Mitchell FM, Buhl AM, Duzie E, Johnson GL (1998) [D-Arg^1^, D-Phe^5^, D-Trp^7,9^,Leu^11^]Substance P acts as a biased agonist toward neuropeptide and chemokine receptors. J Biol Chem 273: 3097–3104 9446627 10.1074/jbc.273.5.3097

[bib17] Jensen R, Jones SW, Folkers K, Gardner JD (1984) A synthetic peptide that is a bombesin receptor antagonist. Nature 309: 61–63 6201745 10.1038/309061a0

[bib18] Jones DA, MacLellan AJ, Cummings J, Ritchie AA, Langdon SP, Smyth JF (1997) Processing of [D-Arg^1^, D-Phe^5^, D-Trp^7,9^, Leu^11^]substance P in xenograft bearing Nu/Nu mice. Peptides 18: 1073–1077 9357069 10.1016/s0196-9781(97)00042-9

[bib19] Langdon SP, Lawrie SS, Hay FG, Hawkes MM, McDonald A, Hayward IP, Schol DJ, Hilgers J, Leonard RC, Smyth JF (1988) Characterization and properties of nine human ovarian adenocarcinoma cell lines. Cancer Res 48: 6166–6172 3167863

[bib20] Langdon S, Sethi T, Ritchie A, Muir M, Smyth J, Rozengurt E (1992) Broad spectrum neuropeptide antagonists inhibit the growth of small cell lung cancer *in vivo*. Cancer Res 52: 4554–4557 1379515

[bib21] Lieber M, Mazzetta J, Nelson-Rees W, Kaplan M, Todaro G (1975) Establishment of a continuous tumour-cell line (panc-1) from a human carcinoma of the exocrine pancreas. Int J Cancer 15: 741–747 1140870 10.1002/ijc.2910150505

[bib22] MacKinnon AC, Armstrong RA, Waters CM, Cummings J, Smyth JF, Haslett C, Sethi T (1999) [Arg^6^, D-Trp^7,9^, *N*^me^Phe^8^]-substance P (6–11) activates JNK and induces apoptosis in small cell lung cancer cells via an oxidant-dependent mechanism. Br J Cancer 80: 1026–1034 10362111 10.1038/sj.bjc.6690458 PMC2363053

[bib23] MacKinnon AC, Waters C, Jodrell D, Haslett C, Sethi T (2001) Bombesin and substance P analogues differentially regulate G-protein coupling to the bombesin receptor. Direct evidence for biased agonism. J Biol Chem 276: 28083–28091 11323408 10.1074/jbc.M009772200

[bib24] Moody TW, Carney DN, Cuttitta F, Quattrocchi K, Minna JD (1985) High affinity receptors for bombesin/GRP-like peptides on human small cell lung cancer. Life Sci 37: 105–113 2409423 10.1016/0024-3205(85)90413-8

[bib25] North WG, Fay MJ, Longo K, Du J (1997) Functional vasopressin V1 type receptors are present in variant as well as classical forms of small-cell carcinoma. Peptides 18: 985–993 9357056 10.1016/s0196-9781(97)00072-7

[bib26] Reubi JC, Schaer JC, Waser B (1997) Cholecystokinin(CCK)-A and CCK-B/gastrin receptors in human tumours. Cancer Res 57: 1377–1386 9102227

[bib27] O'Hara BM, Oskarsson M, Tainsky MA, Blair DG (1986) Mechanism of activation of human ras genes cloned from a gastric adenocarcinoma and a pancreatic carcinoma cell line. Cancer Res 46: 4695–4700 3731120

[bib28] Seckl MJ, Newman RH, Freemont PS, Rozengurt E (1995) Substance P-related antagonists inhibit vasopressin and bombesin but not 5′-3-*O*-(thio)triphosphate-stimulated inositol phosphate production in Swiss 3T3 cells. J Cell Physiol 163: 87–95 7534771 10.1002/jcp.1041630110

[bib29] Sethi T, Herget T, Wu SV, Walsh JH, Rozengurt E (1993) CCKA and CCKB receptors are expressed in small cell lung cancer lines and mediate Ca^2+^ mobilisation and clonal growth. Cancer Res 53: 5208–5213 8221657

[bib31] Sethi T, Langdon S, Smythe J, Rozengurt E (1992) Growth of small cell lung cancer cells: stimulation by multiple neuropeptides and inhibition by broad spectrum neuropeptides antagonists *in vitro* and *in vivo*. Cancer Res 52: 27375–27425 1314136

[bib30] Sethi T, Rozengurt E (1991) Multiple neuropeptides stimulate clonal growth of small cell lung cancer: effects of bradykinin, vasopressin, cholecystokinin, galanin and neurotensin. Cancer Res 51: 3621–3623 1711414

[bib32] Stark LA, Din FVN, Zwacka RM, Dunlop MG (2001) Aspirin-induced activation of the NF-κB signalling pathway: a novel mechanism for aspirin-mediated apoptosis in colon cancer cells. FASEB J 15: 1273–1275 11344111

[bib33] Tallett A, Chilvers ER, MacKinnon AC, Haslett C, Sethi T (1996) Neuropeptides stimulate tyrosine phosphorylation and tyrosine kinase activity in small cell lung cancer cell lines. Peptides 17: 665–673 8804078 10.1016/0196-9781(96)00055-1

[bib34] Woll PJ, Rozengurt E (1988) Two classes of antagonist interact with receptors for the mitogenic neuropeptides bombesin, bradykinin, and vasopressin. Growth Factors 1: 75–83 2483337 10.3109/08977198809000249

